# Otomandibular microsomia: case report

**DOI:** 10.1590/S1808-86942011000100024

**Published:** 2015-10-19

**Authors:** Jozinete Vieira Pereira, Luiz Guedes de Carvalho Neto, Rudyard dos Santos Oliveira, Lúcia de Fátima de Oliveira Costa, Rosemberg de Oliveira Costa

**Affiliations:** 1 Full professor of stomatology, Paraíba Federal University; 2 Paris University. Professor and coordinator of the buccomaxillofacial surgery and trauma unit, Campina Grande Graduate and Research Institute, PB; 3 Dentist, dental surgeon, Paraíba State University; 4 Dentist, dental surgeon, Paraíba State University; 5 Paraíba State University

**Keywords:** maxillofacial abnormalities, facial asymmetry, mandibulofacial dysostosis

## INTRODUCTION

Otomandibular anomalies comprise a heterogeneous group from an embryopathogenic and etiological standpoint; they may be characterized by congenital malformations affecting the mandible and the auditory system. Knowledge about normal embryonic development is an important requirement for the treatment of these malformations.^1^

Otomandibular dysplasias include all disorders that involve ear hypoplasia or agenesis and mandibular hypoplasia. They may be unior bilateral; the latter may be symmetrical or asymmetrical. Other malformations may coexist.^2^

Patients with the classical features of hemifacial microsomia are relatively easy to diagnose medically. An examination by an otorhinolaryngologist and speech therapist soon after birth will demonstrate the extent of hearing loss and the degree of airway involvement. There are, however, subjects that are mildly affected; in these cases, the diagnosis may not be given clinically, and a genetic assessment may be required.^3,^^4^

Traditionally, the diagnosis may be made prenatally by genetic studies of chorionic villus sampling or amniocentesis material, respectively on the 10th to 11th weeks or the 16th to 17th of gestation.^5^ Ultrasound and fetoscopy may also provide the diagnosis. There is a published study in which ultrasound was used to detect the downward-slanting of ears and antimongoloid (downward-slanting) palpebral fissures.^3,^^5^

The post-natal diagnosis is made by DNA analysis of subjects with this syndrome. It is also advisable to study the DNA of parents for genetic counseling purposes.^6^

## CASE REPORT

**Figure 1:**
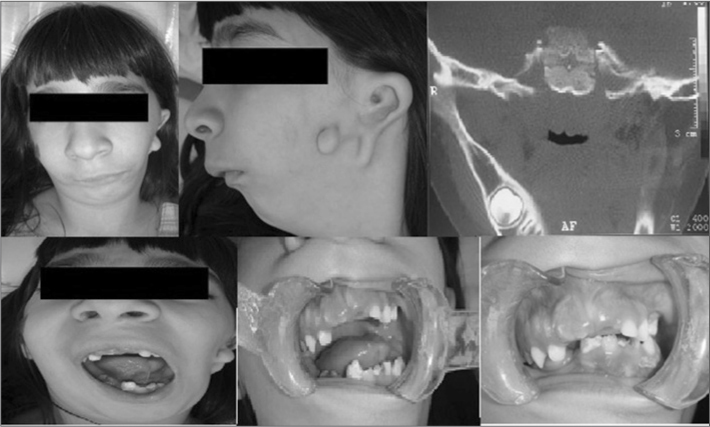
Hemifacial microsomia.

MOM, a white female patient aged 6 years, was brought to the outpatient Buccomaxillofacial Surgery Unit, presenting signs and symptoms suggesting hemifacial microsomia, which was confirmed by the physical examination and tomographic radiography. A lateral view revealed laterognathia of the mandible, midline deviation on the midface, and absence of the left ear (Fig. 1). A left lateral view showed mandibular retrognathism, auricle defects, a bifid auricular lobule, and absence of the outer ear canal (Fig. 1).

The examination of the mouth showed absence of dental elements 51, 61 and 84, midline deviation, limited opening of the mouth, and a posterior left crossbite (Fig. 1).

Computed tomography showed, on the coronal section, absence of the angle, the ascending ramus, the coronoid process and the condylar process of the left mandible (Fig. 1).

The conclusion was that the patient presented signs and symptoms of hemifacial microsomia (or otomandibular microsomia). Corrective surgery and orthopedic-functional therapy were recommended to yield a more harmonic facial conformation. This approach included plastic surgery (to rebuild the ear), a visit to an otorhinolaryngologist for audiometry, buccomaxillofacial surgery for osteogenic distraction, and direct monitoring by a dental surgeon, a medical geneticist and a psychologist.

## DISCUSSION

Patients with craniofacial anomalies are prone to have airway obstruction. A multidisciplinary team is essential for correctly managing the airways of these patients. Micrognathia, abnormal tongue posture, laryngeal hypoplasia, and narrowed larynx and trachea are common findings; these may result in airway obstruction and the obstructive sleep apnea syndrome.^1,^^4,^^6^

Computed tomography of the rhinopharynx is a useful method for defining anatomical airway obstruction.^5^ This method may reveal detail of choanal atresia or other malformations that may result in airway obstruction.^1,^^3,^^5^

Successful treatment of facial asymmetry appears to depend on the severity of the anomaly. The long-term progression may be difficult to predict after growth is complete in extreme cases with muscle deficiencies.^4,^^6^

Thus, only a detailed clinical history of hemifacial microsomia patients, supported by exams (spinal radiograms, echocardiography) and expert consultations (otorhinolaryngologists, ophthalmologists, geneticists, and dental surgeons), may define this syndrome and other associated malformations.^2,^^3,^^4,^^6^

## COMMENTS

Craniofacial anomalies are among the most common human congenital disorders; these conditions require high-cost multiprofessional care. The medical geneticist, one of the team members, is prepared to define the etiology and nosology of these disorders, which is essential for genetic counseling and for gathering epidemiological data. A dental surgeon provides rehabilitation of the stomatognathic system, focusing on the function of this system. Multiple factors are involved in the etiology of these disorders (teratogenic or of genetic origin). These features compound the patient's suffering, because of guilt, the fear of recurrence, and the possible extension to other family members. Thus, genetic and clinical assessments and genetic counseling are paramount when monitoring these individuals.
